# Optimizing Voronoi-based quantifications for reaching interactive analysis of 3D localizations in the million range

**DOI:** 10.3389/fbinf.2023.1249291

**Published:** 2023-08-04

**Authors:** Florian Levet

**Affiliations:** ^1^ CNRS, Interdisciplinary Institute for Neuroscience, IINS, UMR 5297, University of Bordeaux, Bordeaux, France; ^2^ CNRS, INSERM, Bordeaux Imaging Center, BIC, UAR3420, US 4, University of Bordeaux, Bordeaux, France

**Keywords:** single molecule localisation microscopy (SMLM), segmentation, Voronoi–Delaunay tessellation, GPU (CUDA), clustering

## Abstract

Over the last decade, single-molecule localization microscopy (SMLM) has revolutionized cell biology, making it possible to monitor molecular organization and dynamics with spatial resolution of a few nanometers. Despite being a relatively recent field, SMLM has witnessed the development of dozens of analysis methods for problems as diverse as segmentation, clustering, tracking or colocalization. Among those, Voronoi-based methods have achieved a prominent position for 2D analysis as robust and efficient implementations were available for generating 2D Voronoi diagrams. Unfortunately, this was not the case for 3D Voronoi diagrams, and existing methods were therefore extremely time-consuming. In this work, we present a new hybrid CPU-GPU algorithm for the rapid generation of 3D Voronoi diagrams. Voro3D allows creating Voronoi diagrams of datasets composed of millions of localizations in minutes, making any Voronoi-based analysis method such as SR-Tesseler accessible to life scientists wanting to quantify 3D datasets. In addition, we also improve ClusterVisu, a Voronoi-based clustering method using Monte-Carlo simulations, by demonstrating that those costly simulations can be correctly approximated by a customized gamma probability distribution function.

## Introduction

SMLM has induced a major paradigm shift from conventional light microscopy modalities: SMLM datasets are composed of the precise coordinates, in 2D or 3D, of the molecules being acquired. As pixel-based analysis methods were not adapted, these point clouds have required the development of a whole new set of analysis tools based on geometry and spatial organization (Khater et al., 2020). Among those, Voronoi-based methods have emerged as one of the standard in the field, whether it be for clustering (Andronov et al., 2016), segmentation (Levet et al., 2015; Peters et al., 2017) or colocalization (Levet et al., 2019; Ejdrup et al., 2022) and motion (Beheiry. et al., 2015) analysis.

Nevertheless, the rapid adoption of SMLM by life scientists has highlighted an important limitation of existing analysis methods. As datasets composed of millions of localizations are now routinely acquired in biology labs and facilities, it has become obvious that very few methods were capable of scaling to this magnitude, and that especially in 3D. This results in the necessity to define small ROIs composed of 10^4^–10^5^ localizations for analysis, as quantifying a whole dataset in one go is out of reach. For instance, and while 3D analysis with Voronoi diagrams is available for a few years now, it takes several hours to quantify datasets in the 1.10^6^ localization range (Andronov et al., 2018). This unfortunately lacks the interactivity required when analyzing an unknown biological model, as life scientists may need to fine-tune their analysis parameters through several attempts.

In this paper, we propose to optimize two Voronoi-based quantification methods for segmentation and clustering, SR-Tesseler (Levet et al., 2015) and ClusterVisu (Andronov et al., 2016; Andronov et al., 2018), to make them interactive for analyzing 3D SMLM datasets in the millions range. Our contributions are twofold. First, we have developed a new hybrid CPU-GPU algorithm (called Voro3D) for the rapid generation of 3D Voronoi diagrams. Voro3D allows constructing diagrams composed of millions of localizations in a few minutes, achieving state of the art timing for heterogeneous point clouds. Second, we have shown that clustering can be performed without requiring the use of costly Monte Carlo simulations (Andronov et al., 2016; Andronov et al., 2018), as the cell size distribution of randomly placed localizations can be approximated by an analytical function. All these developments are available in our newly released PoCA platform (Levet and Sibarita, 2023).

## Methods

### 3D Voronoi diagram generation

**FIGURE 1:**
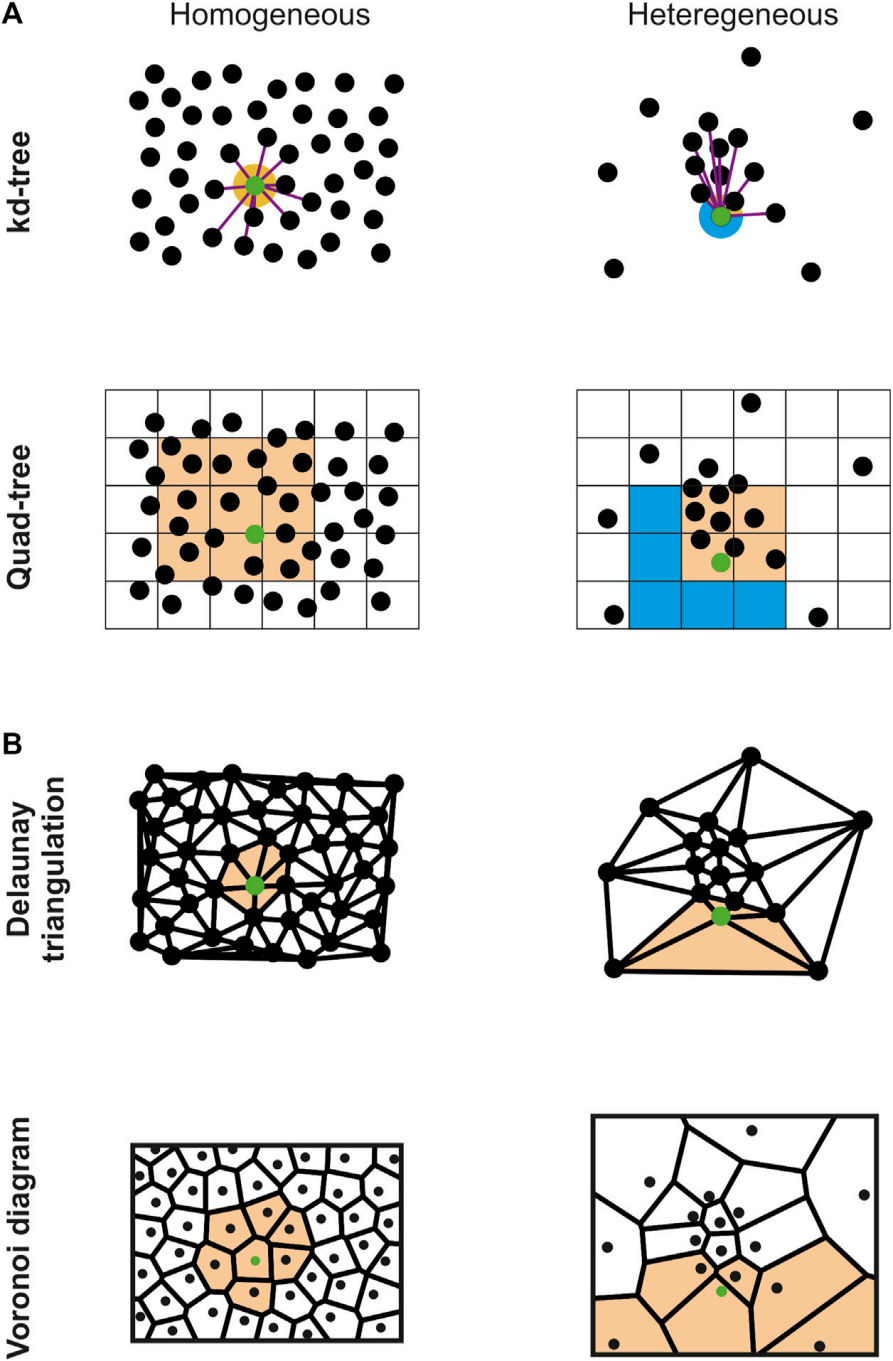
Comparison of the vicinity sampling of a specific point (green) with different spatial subdivision techniques and distributions. **(A)** Supervised spatial subdivision techniques (kd-tree, *n* = 10; quad-tree, side = 25) properly sample the point vicinity (orange) in the homogeneous case (left) but fail to correctly capture some region of the vicinity (blue) for heterogeneous distributions (right). **(B)** On the contrary, unsupervised techniques (Delaunay triangulation and Voronoi diagram) properly sample the point vicinity (orange) for both homogeneous and heterogeneous distributions.

Voronoi diagram is a well-known space-subdividing technique used in dozens of different domains (Atsuyuki et al., 2003) that also met great success in the context of SMLM (Beheiry. et al., 2015; Levet et al., 2015; Andronov et al., 2016; Peters et al., 2017; Andronov et al., 2018; Levet et al., 2019; Ejdrup et al., 2022). Depending on the application, it can be generated with or without its global combinatorial information (i.e., the global mesh geometry) using a variant of the Bower-Watson (Bowyer, 1981; Watson, 1981) or Lloyd (Lloyd, 1982) algorithms. As expected, algorithms generating the global combinatorial information are costlier, thus necessitating access to CPU clusters (Chrisochoides and Nave, 2003; González, 2016) or machines with large number of cores (CGAL, 2020), two equipment that may be out of reach for most biology labs. On the other hand, and when this combinatorial information is unnecessary, each Voronoi cell can be computed individually without requiring synchronization of the global geometry. In this case, each cell only requires to gather its direct neighbors to be generated. Algorithms based on this idea combine supervised spatial subdivisions (subdivision techniques requiring a user-defined parameter for gathering neighbors) with parallelization (Rycroft, 2009; Ray et al., 2018) to speed up the computation speed (Figure 1A). Nevertheless, these implementations are based on the assumption that the originating point clouds are evenly distributed, i.e., that their spatial distribution is homogeneous, which is in general not the case for SMLM data. In Ray et al. (2018), the authors managed to create 3D Voronoi diagrams of millions of points in a few seconds on the GPU. This impressive result was achieved by retrieving the *k*-closest neighbors of each point with a kd-tree, and by using them to brute force the Voronoi cell construction. Nevertheless, this implies that the *k*-nearest neighbors surrounding a point represent a proper sampling of its vicinity, a hypothesis that is only certified in the case of a homogeneous distribution combined with a sufficient number of neighbors (Figure 1A top).

To adapt this process to inhomogeneous point cloud data, we used the Delaunay triangulation, which is the dual of the Voronoi diagram, to compute the Voronoi diagram (Algorithm 1). We integrated a very efficient CPU implementation available in the CGAL (2020) library, that allows computing a 3D Delaunay diagram of 10 million of localizations in around 10 s with a standard computer. Using the connectivity provided by the Delaunay triangulation, all the direct neighbors *p*_*n*_ of a given point *p*, i.e., the points part of the tetrahedrons originating from *p*, can be efficiently retrieved. Contrary to the k-nearest neighbors, *p*_*n*_ is guaranteed to accurately describe the vicinity of *p* in all directions (Figure 1B top). We then modified the algorithm provided in (Ray et al., 2018) to brute force the Voronoi diagram construction on the GPU using *p*_*n*_ (Figure 1B bottom), allowing creating the 3D Voronoi diagrams of inhomogeneous SMLM point cloud data composed of millions of localizations in a few minutes. Implementation discrepancies between CGAL and the GPU code (Ray et al., 2018) forced us to develop a function gathering all the localizations’ direct neighbors in an array (Algorithm 1 line 2). In addition, the Voronoi cells are not returned in (Ray et al., 2018) since their application only required to compute the cells volume, a feature for which it is unnecessary to explicitly create the cells. Consequently, we added a CPU function to create the Voronoi cells from the convex hull of the cells’ points (Barber et al., 1996).

Algorithm 1 Voro3D pseudocode.

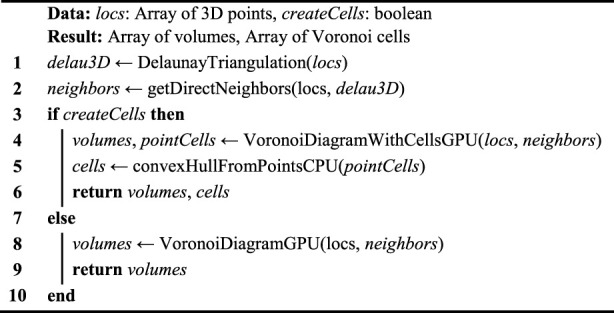

### Voronoi-based clustering

**FIGURE 2:**
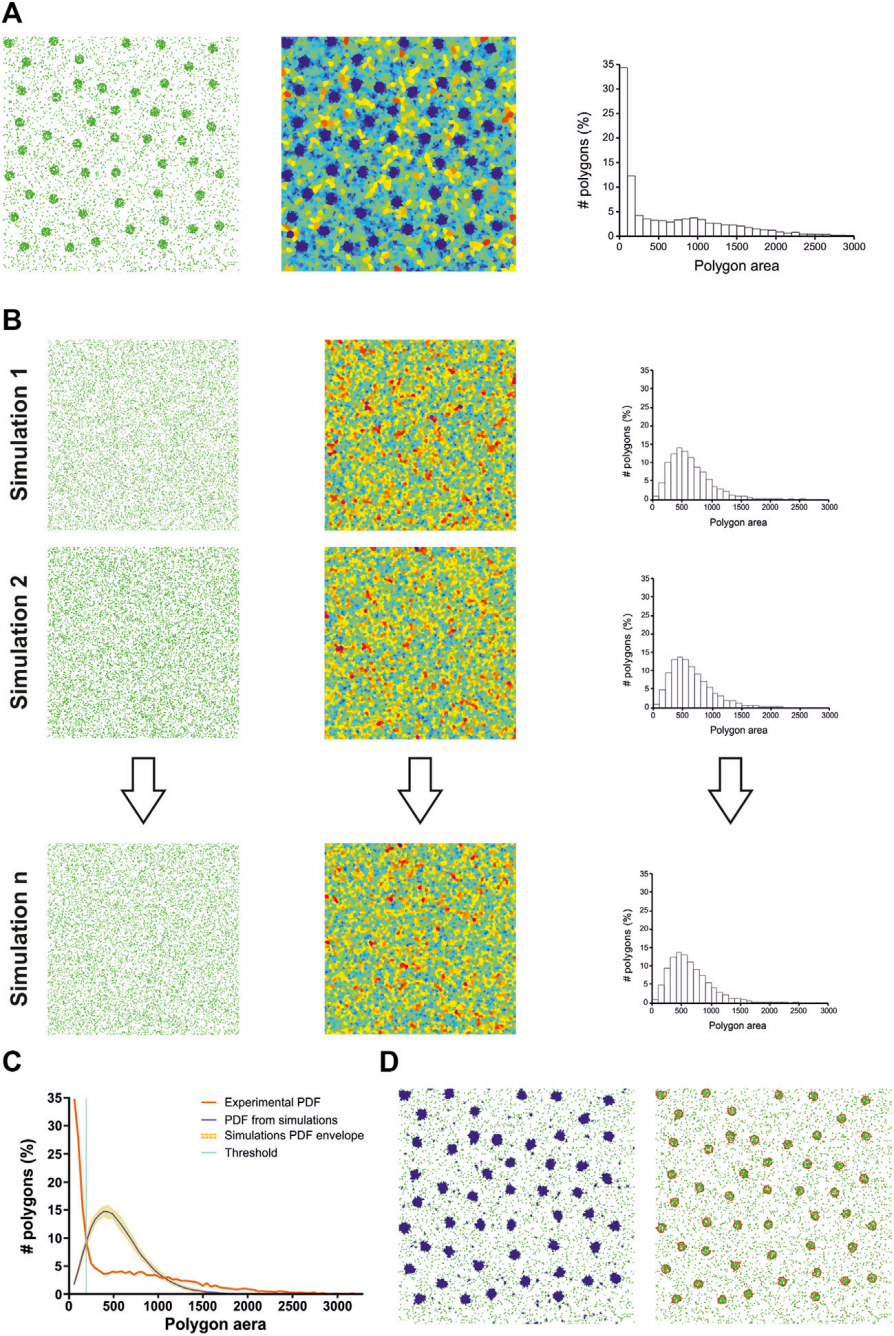
ClusterVisu method. **(A)** Clustered simulation exhibiting 20 clusters (left) with corresponding Voronoi diagram (middle) and cell size distribution (right). **(B)** Simulations of randomly placed points (left) having the same density and area of the clustered simulation with corresponding Voronoi diagrams (middle) and cell size distributions (right). **(C)** Threshold (green) is identified as the intersection between the cell size distribution of the clustered simulation (red) and the mean of the cell size distributions of all the random simulations (blue), with the envelope being displayed in orange. **(D)** Corresponding Voronoi cells selection (left) and clusters (right).

To perform statistical clustering, Andronov et al. compared experimental SMLM datasets with Monte Carlo simulations (Andronov et al., 2016; Andronov et al., 2018) (Figure 2). They generated a user-defined number of simulations having the same number of localizations and volume but exhibiting a random spatial distribution (Figures 2A, B). By comparing the cell size distribution (i.e., volume for three dimensional datasets) of the experimental dataset with the mean cell size distribution of all the simulations, they were able to automatically determine a threshold to segment clusters (Figures 2C, D). Their technique has a strong limitation: as their implementation of the 3D Voronoi diagram was not optimized, and since the global computation time linearly scales with the number of simulations, 3DClusterVisu took up to 6 h for analyzing datasets in the 1.10^6^ localizations range.

Voronoi diagrams of random spatial distributions are called Poisson Voronoi diagrams and have been extensively studied (Hermann et al., 1989; Kumar et al., 1992; Masaharu, 2003; Ferenc and Néda, 2007; Hinde and Miles, 2007; Zaninetti, 2009; González and Einstein, 2011). Of particular interest is the cell size distribution $g\left(y\right)=S$ , with S the cells’ size, which is used in ClusterVisu (Andronov et al., 2016; Andronov et al., 2018). Its normalization $f\left(y\right)=g\left(y\right)/\langle S\rangle$ , with $\langle S\rangle$ the mean of the cells’ size, can be expressed as an analytical function with closed form, but only for the one dimensional case (Ferenc and Néda, 2007). However there exist several probability distribution functions that provide an approximate numerical solution, based on Gamma distributions (Enderlein et al., 1961) having two (*a* and *b*) (Ferenc and Néda, 2007) or three (*a*, *b* and *c*) (Masaharu, 2003; Hinde and Miles, 2007) parameters:

fy=cbacΓacya−1exp−byc

$$f\left(y\right)=\frac{b^{a}}{\Gamma \left(a\right)}y^{a-1}\exp\left(-by\right)$$

with *a* and *c* two shape parameters and *b* a scale parameter. From all the proposed distributions, we used the ones from Ferenc and Néda (2007):

f2Dy=3431572πy52exp−72y, (1)

$$f_{3D}\left(y\right)=\frac{3125}{24}y^{4}\exp\left(-5y\right)$$ (2)

The error between the cell size distribution of simulated datasets and this analytical distribution has been quantified to be less than 0.04% and 1%, for 2D and 3D, respectively (Ferenc and Néda, 2007).

Nevertheless, blinking of the molecules is not taken into account by this numerical approximation. Monte Carlo simulations, on the other hand, should be able to integrate this blinking behavior in the cell size distribution, as long as the simulator provides a realistic simulation of blinking, a task known to be difficult. Another difference between the numerical approximation and Monte Carlo simulations is the cell size distribution envelope provided by running several simulations (Figure 2C). This envelope helps to assess how much an experimental dataset deviates from a random distribution of points. Another potential solution to quantify this deviation is to compute the Ripley’s functions (Ripley, 1977; Nieves et al., 2023).

## Results

**FIGURE 3:**
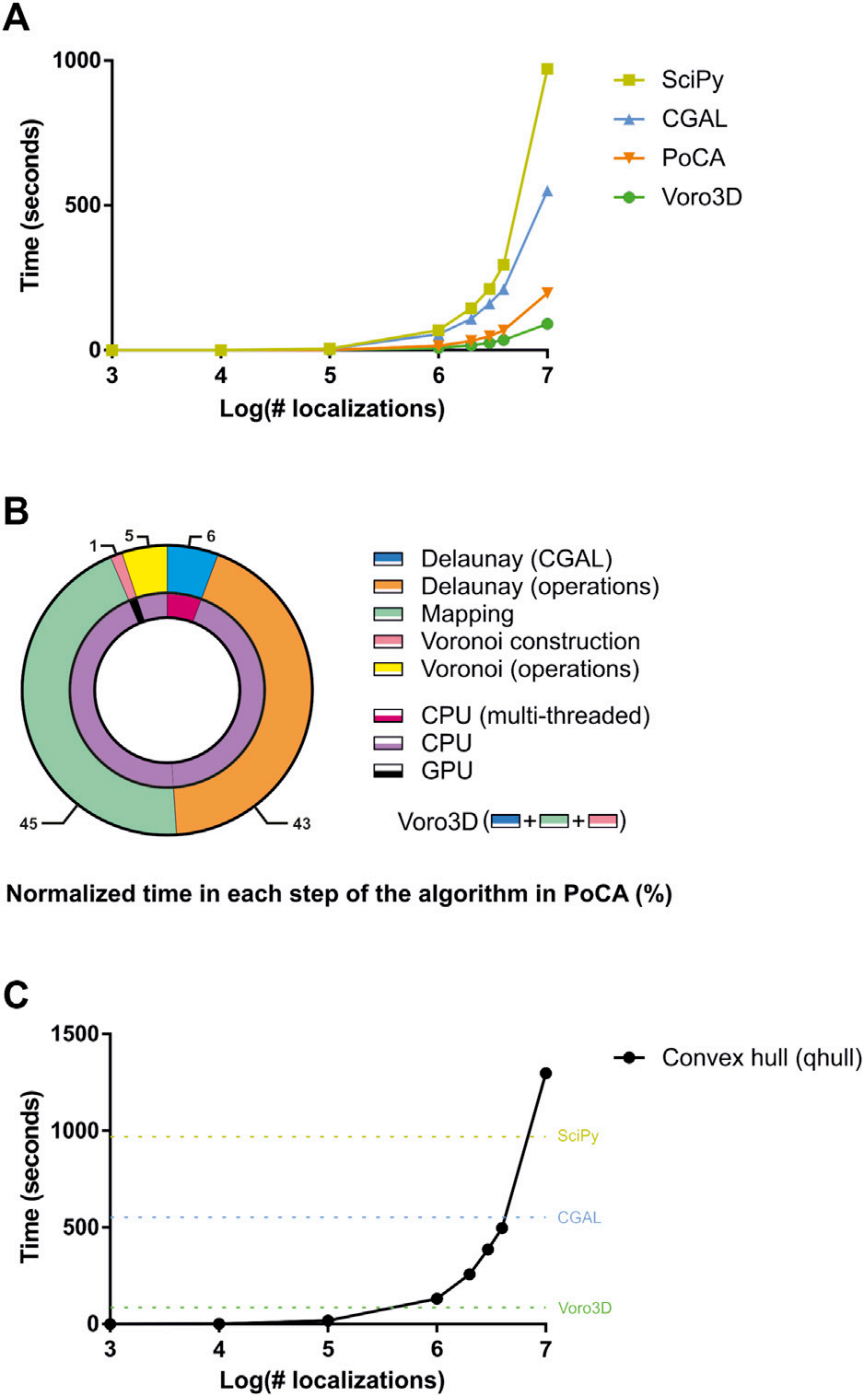
Comparison of timings for different algorithms. **(A)** Comparison of timings for SciPy, CGAL, PoCA and Voro3D for datasets ranging from 1,000 to 10,000,000 localizations. **(B)** Normalized timings for each step of the 3D Voronoi diagram generation implemented in PoCA. **(C)** Timings for computing the convex hull of the Voronoi cells for datasets ranging from 1,000 to 10,000,000 localizations. For comparison, the timings for generating the 3D Voronoi diagram for 10,000,000 localizations with Scipy (dark yellow), CGAL (blue) and Voro3D (green) are plotted.

Voro3D is available in our newly released SMLM analysis platform called PoCA (Levet and Sibarita, 2023). While PoCA is developed in C++ and can therefore be executed on any computer, Voro3D is implemented in Cuda (Nickolls et al., 2008) and therefore requires a computer equipped with a Nvidia graphics card (https://www.nvidia.com/). To our knowledge, the only two libraries implementing 3D Voronoi diagrams for heterogeneous point clouds that are directly accessible are SciPy (Virtanen et al., 2020) (Supplementary Material) and CGAL (2020), which was included in one of our precedent software platform (Levet et al., 2019). We therefore compared the execution time of Voro3D versus SciPy and CGAL for datasets exhibiting a random spatial distribution and ranging from 10^3^ to 10^7^ localizations (Figure 3A). Voro3D is consistently faster than the other two solutions. It achieves generating a 3D Voronoi diagram from a dataset composed of 10 million localizations in close to 1 min and a half, a time suitable with an interactive analysis. Importantly, the implementation of Voro3D available in PoCA takes longer as more operations are performed. In addition to the three steps of the pseudocode (Algorithm 1), there are two additional steps pertaining to computational operations performed on the Delaunay triangulation and the Voronoi diagram, such as computation of the tetrahedra volume or creation of the OpenGL rendering buffers (Figure 3B). Time ratio between the different steps shows the extreme efficiency of both the Delaunay triangulation generation (multithreaded on the CPU, 10.4 s for 10 million localizations) and the construction of the Voronoi cells (GPU, 1.55 s for 10 million localizations). Most of the time is spent on operations performed on CPU without multithreading (92%), such as computational operations on the Delaunay triangulation (43%) or gathering the direct neighbors for the Voronoi cell construction (45%). Finally, the Voronoi GPU code (Ray et al., 2018) computes the Voronoi cells as a combination of tetrahedra, i.e., Voronoi cells are not explicitly constructed and returned. We therefore added an optional function to compute the Voronoi cells based on the convex hull (Barber et al., 1996) (Algorithm 1). This function is time consuming as it is single-threaded and non-optimized (Figure 3C).

**FIGURE 4:**
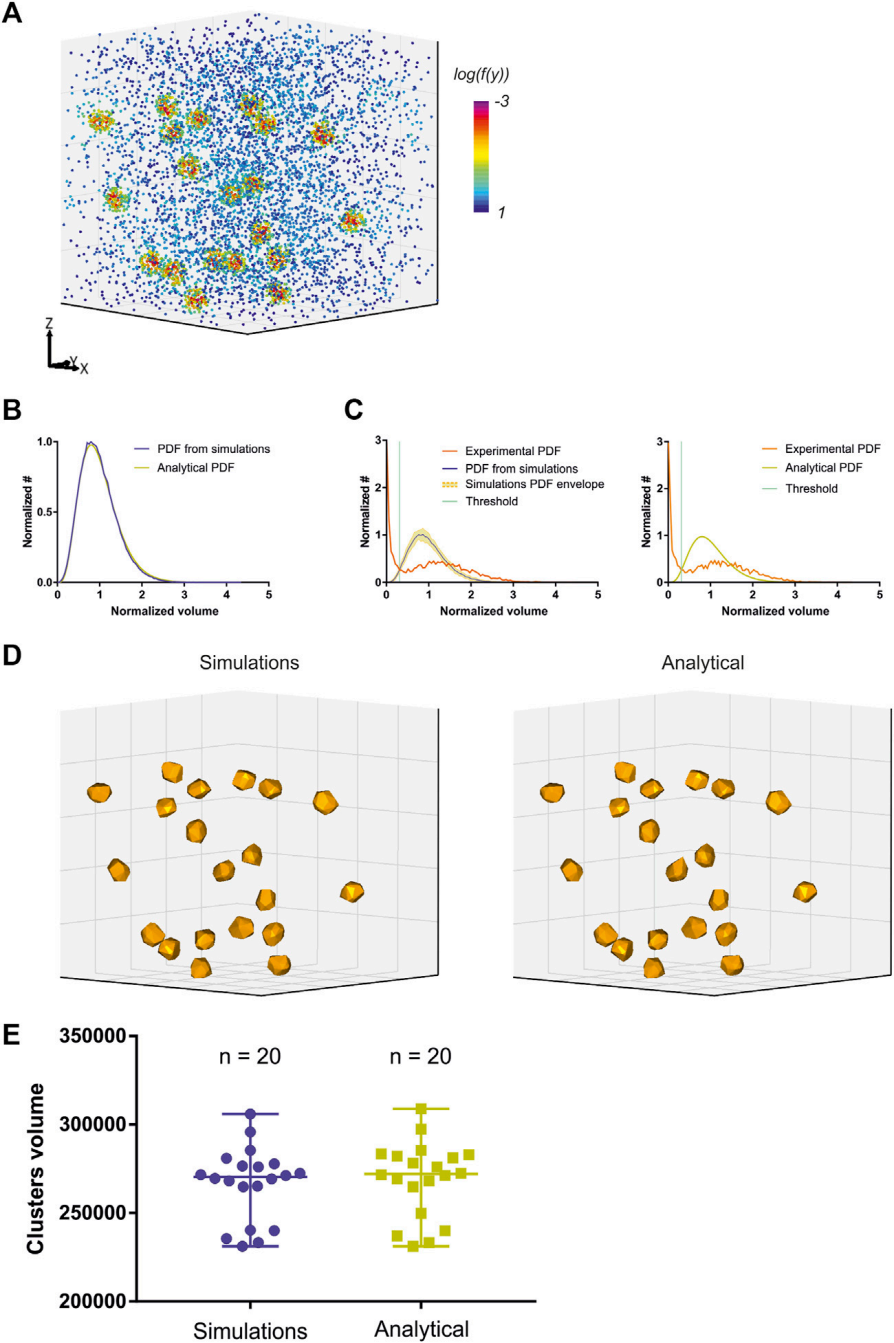
Comparison of clustering with Monte-Carlo simulations and a customized gamma distribution on simulations. **(A)** Simulation of a 3D dataset exhibiting 20 clusters. **(B)** Comparison of the distribution of normalized Voronoi cells volume between Monte-Carlo simulations (blue) and Eq. 2 (dark yellow). **(C)** Computed threshold (green) with Monte-Carlo simulations (left) and Eq. 2 (right). **(D)** Segmented clusters with Monte-Carlo simulations (left) and Eq. 2 (right). **(E)** Comparison of the segmented cluster volumes with Monte-Carlo simulations (blue) and Eq. 2 (dark yellow).

**FIGURE 5:**
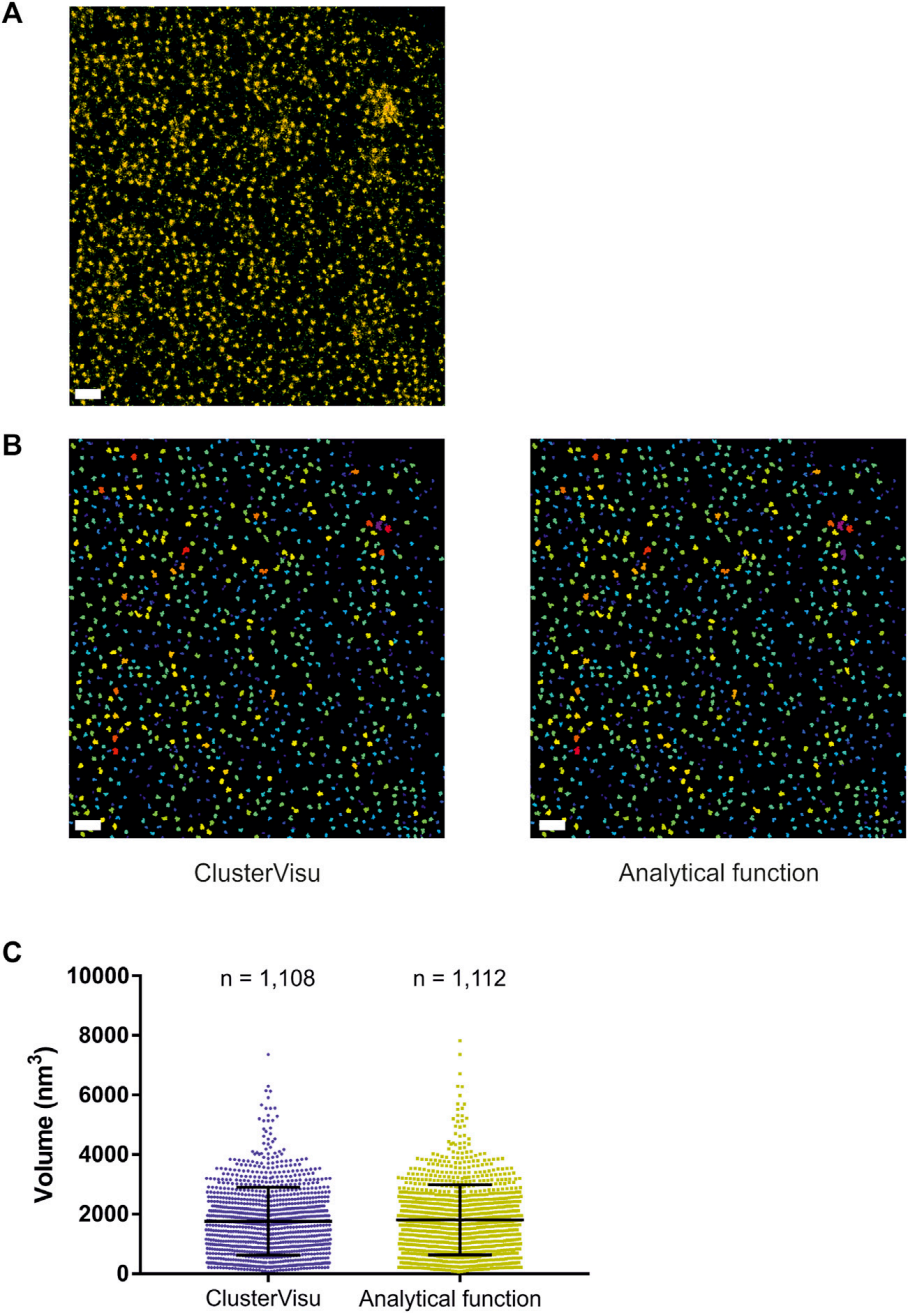
Comparison of clustering with Monte-Carlo simulations and a customized gamma distribution on an experimental dataset. **(A)** Localization dataset of *Xenopus laevis* nuclear pore complexes stained with WGA-ATTO520, scale bar = 500 nm. **(B)** Segmented clusters obtained with ClusterVisu (left) and Eq. 2 (right), scale bar = 500 nm. **(C)** Comparison of the segmented cluster volumes with Monte-Carlo simulations (blue) and Eq. 2 (dark yellow).

While Voro3D already greatly improves the computation time of 3DClusterVisu (15 min with Voro3D versus 6 h for the original 3DClusterVisu (Andronov et al., 2018) for a dataset composed of 780,000 localizations), using an analytical function approximating the cell size distribution eliminates any additional computational time. Therefore, to demonstrate that Eq. 2 is a good approximation for 3DClusterVisu (Andronov et al., 2018) we first generated a simulated dataset composed of 20 clusters (Figure 4A, referred as experimental), and then computed fifty Monte Carlo simulations having the same localization density but with a random spatial distribution (referred as simulations). The cell volume probability distribution function (PDF) of experimental and simulations were both normalized to be comparable with the analytical PDF (Figures 4B, C). Importantly, we also removed Voronoi cells that were on the border of the dataset volume (i.e., they were cut by the dataset bounding volume) to prevent any edge effect. We found that the simulations and analytical PDFs were not significantly different (Figure 4B, Wilcoxon rank test *p*-value = 0.1211), a property that also transfers to the computed thresholds (Figure 4C, T_simulations_ = 0.307, T_analytical_ = 0.312). Finally, objects created with these thresholds exhibited similar shapes and volumes (Figures 4D, E) and their volume distributions were not significantly different (Kolmogorov-Smirnov test *p*-value = 0.818). We also compared the volume of each object in the simulations with its counterpart in the analytical and quantified the mean difference to be less than 1%. Finally, we also compared clustering between Monte-Carlo simulations and Eq. 2 on an experimental dataset of *Xenopus laevis* nuclear pore complexes stained with WGA-ATTO520 (Löschberger, 2021) available for download on ShareLoc (Ouyang et al., 2022) (Figure 5A). Similarly to the simulations, the number of segmented clusters is very similar (ClusterVisu, *n* = 1,108; Eq. 2, *n* = 1,112; Figure 5B) and their distributions of volume are not significantly different (Kolmogorov-Smirnov test *p*-value = 0.788, Figure 5C).

## Discussion

As 3D SMLM is increasingly adopted by biology labs by ways of imaging facilities, the bottleneck has shifted from not having the technology to image to not being able to analyze and extract meaningful quantifications from the acquired datasets. In the context of 2D SMLM analysis, Voronoi diagrams has become a method of reference (Levet et al., 2015; Andronov et al., 2016; Peters et al., 2017; Andronov et al., 2018; Levet et al., 2019) thanks in part to its rapidity that gave life scientists interactive feedbacks on quantifications. Unfortunately, 3D Voronoi analysis was extremely time-consuming, preventing its use for datasets composed of millions of 3D localizations.

In this paper, we presented a novel hybrid CPU-GPU algorithm called Voro3D for the fast computation of 3D Voronoi diagrams. This algorithm achieves state of the art computation time for point clouds exhibiting heterogeneous spatial distribution. While its implementation in PoCA is slightly more computationally expensive, it is still compatible with an interactive analysis. Voro3D also improves the computation time of any method based on Voronoi diagrams, such as ClusterVisu (Andronov et al., 2016; Andronov et al., 2018). Nevertheless, we also demonstrated that the main component of ClusterVisu, the cell size probability distribution function, can be correctly approximated by a customized gamma distribution. This results in the capability of computing clustering of SMLM datasets with no other additional cost than the Voronoi diagram computation.

From the three steps of the Voro3D algorithm, inspection of the computation time has shown that the majority of the computation time is spent on transferring the localizations’ neighbors from the Delaunay triangulation to the Voronoi diagram (Delaunay computation: 12%, transferring neighbors: 86%, Voronoi computation: 2%). This transfer step is currently required because of discrepancies between the CGAL and Cuda implementations. Multi-threading this step could reduce even more the Voro3D computation time. Similarly, construction of the Voronoi cells is currently time consuming as it is a single-thread CPU process. Constructing the cell at the same time than the tetrahedral decomposition happening in the GPU could massively improve its computation time.

Voro3D is natively included in PoCA and our implementation of ClusterVisu (with Monte Carlo simulations and our analytical approximation) is available as a PoCA’s plugin.

## Data Availability

Publicly available datasets were analyzed in this study. This data can be found here: https://zenodo.org/record/7182237.

## References

[B1] Andronov L. Michalon J. Ouararhni K. Orlov I. Hamiche A. Vonesch J. L. (2018). 3DClusterViSu: 3D clustering analysis of super-resolution microscopy data by 3D voronoi tessellations. Bioinformatics 34, 3004–3012. 10.1093/bioinformatics/bty200 29635310

[B2] Andronov L. Orlov I. Lutz Y. Vonesch J. Klaholz B. P. (2016). ClusterViSu, a method for clustering of protein complexes by Voronoi tessellation in super-resolution microscopy. Nat. Publ. Gr. 6, 24084–24089. 10.1038/srep24084 PMC4828638 27068792

[B3] Atsuyuki O. Barry. B. Kokichi S. Sung Nok C. (2003). Spatial tessellations: Concepts and applications of Voronoi diagrams. Hoboken: Wiley.

[B4] Barber C. B. Dobkin D. P. Huhdanpaa H. (1996). The quickhull algorithm for convex hulls. ACM Trans. Math. Softw. 22, 469–483. 10.1145/235815.235821

[B5] Beheiry. M. E. Dahan M. Masson J. B. (2015). InferenceMAP: Mapping of single-molecule dynamics with bayesian inference. Nat. Methods 2015 127 (12), 594–595. 10.1038/nmeth.3441 26125589

[B6] Bowyer A. (1981). Computing dirichlet tessellations. Comput. J. 24, 162–166. 10.1093/comjnl/24.2.162

[B7] CGAL (2020). The CGAL project {CGAL} user and reference manual. CGAL editorial board.

[B8] Chrisochoides N. Nave D. (2003). Parallel Delaunay mesh generation kernel. Int. J. Numer. Methods Eng. 58, 161–176. 10.1002/nme.765

[B9] Ejdrup A. L. Lycas M. D. Lorenzen N. Konomi A. Herborg F. Madsen K. L. (2022). A density-based enrichment measure for assessing colocalization in single-molecule localization microscopy data. Nat. Commun. 2022 131 (13), 4388–4410. 10.1038/s41467-022-32064-y PMC9334352 35902578

[B10] Enderlein G. Hogg R. V. Craig A. T. (1961). Introduction to mathematical statistics. The macmillan company, New York, 1 Print 1959, 245 seiten, $ 6,75. Biom. Z. 3, 145–146. 10.1002/bimj.19610030210

[B11] Ferenc J. S. Néda Z. (2007). On the size distribution of Poisson Voronoi cells. Phys. A Stat. Mech. its Appl. 385, 518–526. 10.1016/j.physa.2007.07.063

[B12] González D. L. Einstein T. L. (2011). Voronoi cell patterns: Theoretical model and applications. Phys. Rev. E. 84, 051135. 10.1103/PhysRevE.84.051135 22181396

[B13] González R. E. (2016). Paravt: Parallel Voronoi tessellation code. Astron. Comput. 17, 80–85. 10.1016/j.ascom.2016.06.003

[B14] Hermann H. Wendrock H. Stoyan D. (1989). Cell-area distributions of planar Voronoi mosaics. Metallography 23, 189–200. 10.1016/0026-0800(89)90030-x

[B15] Hinde A. L. Miles R. E. (2007). Monte Carlo estimates of the distributions of the random polygons of the voronoi tessellation with respect to a Poisson process. Taylor Francis group 10, 205–223. 10.1080/00949658008810370

[B16] Khater I. M. Nabi I. R. Hamarneh G. (2020). A review of super-resolution single-molecule localization microscopy cluster analysis and quantification methods. Patterns 1, 100038. 10.1016/j.patter.2020.100038 33205106 PMC7660399

[B17] Kumar S. Kurtz S. K. Banavar J. R. Sharma M. G. (1992). Properties of a three-dimensional Poisson-voronoi tesselation: A Monte Carlo study. J. Stat. Phys. 67, 523–551. 10.1007/bf01049719

[B18] Levet F. (2015). SR-tesseler: A method to segment and quantify localization-based super-resolution microscopy data. Nat. Methods 12. 10.1038/nmeth.3579 26344046

[B19] Levet F. Julien G. Galland R. Butler C. Beghin A. Chazeau A. (2019). A tessellation-based colocalization analysis approach for single-molecule localization microscopy. Nat. Commun. 10, 2379–2412. 10.1038/s41467-019-10007-4 31147535 PMC6542817

[B20] Levet F. Sibarita J.-B. (2023). PoCA: A software platform for point cloud data visualization and quantification. Nat. Methods 20, 1–2. 10.1038/s41592-023-01811-4 36869121

[B21] Lloyd S. P. (1982). Least squares quantization in PCM. IEEE Trans. Inf. Theory 28, 129–137. 10.1109/tit.1982.1056489

[B22] Löschberger A. (2021). *Xenopus laevis* nuclear pore complex stained with WGA-ATTO520. 10.5281/ZENODO.7182237

[B23] Masaharu T. (2003). Statistical distributions of Poisson Vorono¨ı cells in two and three dimensions.

[B24] Nickolls J. Buck I. Garland M. Skadron K. (2008). Scalable parallel programming with CUDA. Queue 6, 40–53. 10.1145/1365490.1365500

[B25] Nieves D. J. Pike J. A. Levet F. (2023). A framework for evaluating the performance of SMLM cluster analysis algorithms. Nat. Methods 2023 202 (20), 259–267. 10.1038/s41592-022-01750-6 36765136

[B26] Ouyang W. Bai J. Singh M. K. (2022). ShareLoc — An open platform for sharing localization microscopy data. Nat. Methods 2022 1911 (19), 1331–1333. 10.1038/s41592-022-01659-0 36271059

[B27] Peters R. Benthem Muñiz M. Griffié J. Williamson D. J. Ashdown G. W. Lorenz C. D. (2017). Quantification of fibrous spatial point patterns from single-molecule localization microscopy (SMLM) data. Bioinformatics 33, 1703–1711. 10.1093/bioinformatics/btx026 28108449

[B28] Ray N. Sokolov D. Lefebvre S. Lévy B. (2018). Meshless voronoi on the GPU. ACM Trans. Graph. 37, 1–12. 10.1145/3272127.3275092

[B29] Ripley B. D. (1977). Modelling spatial patterns. J. R. Stat. Soc. Ser. B 39, 172–192. 10.1111/j.2517-6161.1977.tb01615.x

[B30] Rycroft C. H. (2009). VORO++: A three-dimensional voronoi cell library in C++. Chaos Interdiscip. J. Nonlinear Sci. 19 (4), 041111. 10.1063/1.3215722 20059195

[B31] Virtanen P. Gommers R. Oliphant T. E. (2020). SciPy 1.0: Fundamental algorithms for scientific computing in Python. Methods 2020 173 (17), 261–272. 10.1038/s41592-019-0686-2 PMC7056644 32015543

[B32] Watson D. F. (1981). Computing the n-dimensional Delaunay tessellation with application to Voronoi polytopes. Comput. J. 24, 167–172. 10.1093/comjnl/24.2.167

[B33] Zaninetti L. (2009). Poissonian and non-Poissonian Voronoi diagrams with application to the aggregation of molecules. Phys. Lett. A 373, 3223–3229. 10.1016/j.physleta.2009.07.010

