# Supplementary material for: Optimizing Voronoi-based quantifications for reaching interactive analysis of 3D localizations in the million range
Source: Front Bioinform. 2023 Aug 4;3:1249291. doi: 10.3389/fbinf.2023.1249291 (PMC10436483; doi:10.3389/fbinf.2023.1249291)
Supplement: Supplementary file 1 [file DataSheet1.pdf]

Optimizing Voronoi-based quantifications for reaching interactive analysis of 3D localizations in the million range

Florian Levet[1, 2]

[1]Univ. Bordeaux, CNRS, Interdisciplinary Institute for Neuroscience, IINS, UMR 5297, F-33000 Bordeaux, France

[2]Univ. Bordeaux, CNRS, INSERM, Bordeaux Imaging Center, BIC, UAR3420, US 4, F-33000 Bordeaux, France

Supplementary Material

3D Voronoi diagram generation with SciPy. This Python script was used to evaluate timings of SciPy for generating 3D Voronoi diagrams.

```
import numpy as np
import time
from scipy.spatial import Voronoi
import random

def generate_random_point_cloud(nb_points: int):
    w, h, d = 1000, 1000, 1000
    xs, ys, zs = [], [], []
    for n in range(0, nb_points):
        xs.append(random.uniform(0, w))
        ys.append(random.uniform(0, h))
        zs.append(random.uniform(0, d))
    return xs, ys, zs

nb_points = 780000
xs, ys, zs = generate_random_point_cloud(nb_points)
points = np.vstack([np.asarray(xs), np.asarray(ys), np.asarray(zs)]).T
print(points.shape)
start_time = time.time()
vor = Voronoi(points)
print("--- %s seconds ---" % (time.time() - start_time))
```
